# Short‐term patient‐reported outcomes following rectus femoris versus quadriceps tendon and hamstring tendon autografts for ACL reconstruction with concomitant anterolateral procedure: A prospective comparative study

**DOI:** 10.1002/jeo2.70901

**Published:** 2026-09-17

**Authors:** Tomas Pineda, Nathan H. Varady, Christophe Jacquet, Grigore Mihai, Julien Druel, Matthieu Ollivier

**Affiliations:** ^1^ Facultad de Medicina, Hospital del Trabajador Universidad Andres Bello Santiago Chile; ^2^ Facultad de Medicina, Hospital el Carmen Universidad Finis Terrae Santiago Chile; ^3^ Department of Sports Medicine Hospital for Special Surgery New York New York USA; ^4^ Assistance Publique Hôpitaux de Marseille Institute for Locomotion Marseille France; ^5^ Département of Biomechanics, CNRS, ISM Aix‐Marseille University Marseille France

**Keywords:** anterior cruciate ligament reconstruction, donor site morbidity, hamstring, patient‐reported outcomes, quadriceps autograft, rectus femoris autograft

## Abstract

**Purpose:**

To compare short‐term patient‐reported outcomes following anterior cruciate ligament reconstruction (ACLR) with concomitant anterolateral procedure, using rectus femoris (RF), quadriceps tendon (QT) and hamstring tendons (HS) autografts. It was hypothesized that short‐term patient‐reported outcomes would not differ significantly across graft strategies at 1‐year follow‐up.

**Methods:**

A prospective comparative non‐randomized study was conducted, including 90 consecutive patients undergoing primary ACLR between May 2022 and June 2023 (30 per group). Outcomes were assessed at a median follow‐up of 15.0 months (range: 12–22) using the Knee injury and Osteoarthritis Outcome Score (KOOS) Pain and Quality of Life (QoL) subscales, EuroQOL 5‐dimensions 5‐level index (EQ‐5D‐5L), global satisfaction (0–100) and the Patient and Observer Scar Assessment Scale (POSAS). Continuous variables were compared using one‐way analysis of variance with Bonferroni‐corrected post hoc comparisons and categorical variables using *χ* ^2^ tests. A multivariable logistic regression model was used to assess predictors of POSAS ≤ 20.

**Results:**

Of the 90 consecutive patients initially included, one patient in the RF group was excluded following graft failure and revision surgery prior to outcome assessment, leaving 89 patients available for analysis. Mean global satisfaction was 86.5 ± 10.9, KOOS Pain 89.1 ± 13.2, KOOS QoL 71.4 ± 22.2 and EQ‐5D‐5L Index 0.85 ± 0.15. No statistically significant differences were observed between graft types in KOOS QoL (*p* = 0.344), KOOS Pain (*p* = 0.716), EQ‐5D‐5L (*p* = 0.386) or POSAS (*p* = 0.748). Global satisfaction did not differ significantly across graft groups (*p* = 0.074).

**Conclusion:**

No statistically significant differences in short‐term patient‐reported outcomes were observed among RF, QT and HS autografts at 1‐year follow‐up. Graft strategy was not associated with differences in short‐term postoperative patient‐reported outcomes within the available sample.

**Level of Evidence:**

Level III.

## Graphical abstract

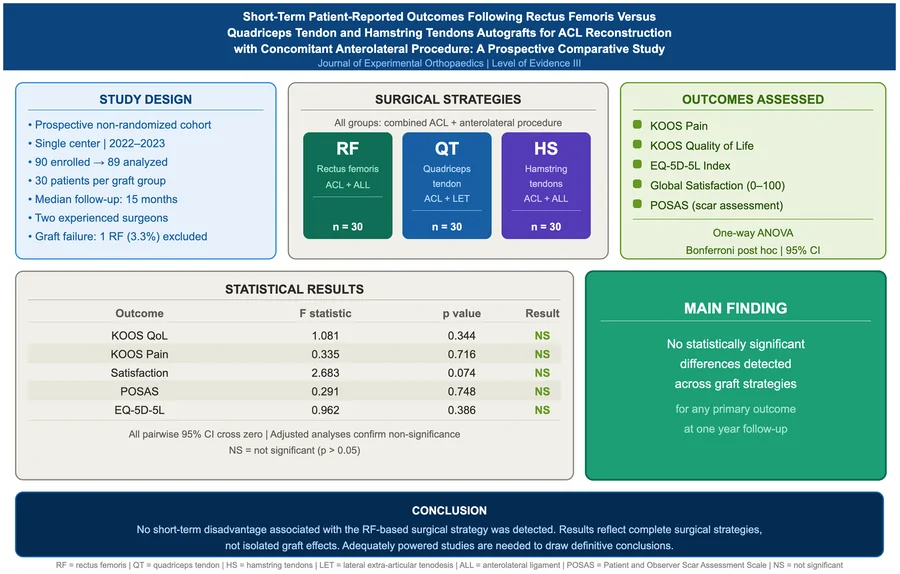

**Abbreviations**
ACLR: anterior cruciate ligament reconstruction
CI: confidence interval
HS: hamstring tendon
KOOS: Knee injury and Osteoarthritis Outcome Score
OR: odds ratio
POSAS: Patient and Observer Scar Assessment Scale
QoL: Quality of Life
QT: quadriceps tendon
RF: rectus femoris

## INTRODUCTION

Graft selection in anterior cruciate ligament reconstruction (ACLR) has progressively expanded beyond purely biomechanical and survivorship considerations to incorporate patient‐oriented factors [7, 12, 20, 22, 24]. While quadriceps tendon (QT) and hamstring tendons (HS) autografts remain established options, interest in the rectus femoris (RF) tendon as an alternative autograft has recently increased [3, 8, 10, 14, 20, 21, 23, 29]. The RF autograft offers several theoretical and technical advantages over established constructs. Its harvest yields a graft of sufficient length to reconstruct both the ACL and the anterolateral ligament (ALL) through small incisions using a single construct, eliminating the need for additional graft harvest from a separate donor site. Furthermore, only the superficial layer of the quadriceps muscle is harvested, leaving the deeper layers intact and potentially reducing donor‐site morbidity. Despite this growing interest among surgeons, evidence specifically addressing short‐term postoperative patient‐reported outcomes following RF autograft ACLR remains scarce.

Most comparative studies in ACLR have focused on objective endpoints, including strength recovery, joint stability, graft survival and return‐to‐sport rates [1, 4, 7, 13, 15, 17, 26]. Although these measures are essential, they may not fully capture the patient experience during the early postoperative period. Pain, knee‐related Quality of Life (QoL), global satisfaction and scar perception may meaningfully influence how patients judge surgical success. However, these domains are less consistently reported in graft selection research [9, 30]. Even among available recent prospective comparative evidence, studies have largely been restricted to other autograft options, with outcome assessment centred on objective measures of laxity and strength recovery rather than subjective recovery domains [6]. As newer graft options are incorporated into clinical practice, understanding their specific impact on subjective patient‐reported domains is necessary before broader implementation.

Therefore, the purpose of this study was to compare short‐term patient‐reported outcomes following ACL reconstruction using RF, QT and HS autografts. It was hypothesized that short‐term patient‐reported outcomes, including pain, knee‐related QoL, global satisfaction and scar perception, would not differ significantly among graft types at 1‐year follow‐up.

## MATERIALS AND METHODS

### Study design and patient population

A prospective, comparative, non‐randomized cohort study was conducted at a single centre between May 2022 and June 2023. Ninety consecutive patients undergoing primary ACL reconstruction were prospectively included. Patients with prior ligament surgery on the index knee or undergoing revision ACL reconstruction were excluded. Additional procedures expected to substantially influence postoperative recovery (e.g., osteotomy or cartilage restoration procedures) were also excluded. Patients were allocated to one of three graft groups, RF, QT or HS, until 30 patients per group were reached. Enrolment across graft groups occurred concurrently throughout the study period rather than sequentially. Graft selection was determined by the operating surgeon in discussion with the patient. Allocation was not randomized in accordance with institutional review board requirements, given the emerging nature of the RF autograft and the limited clinical evidence available at the time of study initiation. All participating surgeons performed reconstructions with all three graft types in comparable proportions.

All patients underwent standardized surgical techniques according to graft type and followed an identical postoperative rehabilitation protocol, with no graft‐specific restrictions. The present analysis focused on short‐term patient‐reported outcomes, aiming to evaluate short‐term postoperative outcomes relevant to daily function, pain, satisfaction and cosmetic perception, rather than return‐to‐sport performance. Patient‐reported outcome measures (PROMs) were collected without blinding to graft type, as allocation was known to both patients and treating surgeons. Institutional review board approval was obtained (Cse_pads25_044_dgr).

### Outcome measures

Knee‐related QoL was assessed using the Knee injury and Osteoarthritis Outcome Score (KOOS) Quality of Life subscale (KOOS‐QoL), and pain was evaluated using the KOOS Pain subscale. Generic health status was measured with the EuroQol 5‐dimension 5‐level index (EQ‐5D‐5L). Global satisfaction was recorded using a 0–100 scale. Scar perception was assessed using the Patient and Observer Scar Assessment Scale (POSAS) [5].

For clinical interpretability, a priori near‐ceiling thresholds were applied as a secondary descriptive analysis to identify patients achieving high‐level postoperative status: KOOS‐Pain ≥90, KOOS‐QoL ≥90, satisfaction ≥90 and POSAS ≤ 20. These thresholds were not externally validated.

### Surgical technique

All procedures were performed by two experienced knee surgeons (M. O. and C. J.) using standardized techniques specific to each graft configuration.

In the QT group, ACLR was performed using a QT autograft harvested with a patellar bone block [19]. In addition, a lateral extra‐articular tenodesis (LET) was performed. In the HS group, the ACL was reconstructed using a tripled semitendinosus graft. The gracilis tendon was harvested and used for reconstruction of the ALL, while preserving the distal hamstring insertions [28]. In the RF group, a RF autograft was harvested and used to reconstruct both the ACL and the ALL using a single femoral tunnel located proximal to the lateral epicondyle [29]. This technique allowed combined intra‐ and extra‐articular reconstruction using a single graft construct.

### Postoperative rehabilitation protocol

All patients followed a standardized, progressive rehabilitation programme under the supervision of a physiotherapist. Immediate postoperative management emphasized early restoration of full knee extension, progressive recovery of flexion range of motion and control of joint effusion. Partial weight‐bearing with crutches was allowed as tolerated during the initial phase and advanced according to quadriceps control and gait normalization.

Closed‐chain strengthening exercises were initiated early, followed by progressive introduction of open‐chain quadriceps strengthening within a controlled range of motion. Neuromuscular training and proprioceptive exercises were incorporated once adequate strength and dynamic control were achieved. Running was generally permitted after restoration of symmetrical strength and absence of effusion, typically around 3–4 months postoperatively, with gradual progression toward sport‐specific drills. Return‐to‐sport clearance was based on functional testing and isokinetic symmetry criteria rather than time alone.

### Statistical analysis

Continuous variables are reported as mean ± standard deviation. Normality was assessed using Shapiro–Wilk tests and visual inspection of Q–Q plots. Given the sample size of approximately 30 per group, one‐way analysis of variance was used for all continuous comparisons. Post hoc pairwise comparisons were performed using Bonferroni correction, with mean differences and 95% confidence intervals (CIs) reported. Effect sizes were calculated using Cohen's *d*. Categorical variables, including high‐threshold outcomes (e.g., satisfaction ≥90 and KOOS thresholds), were compared using *χ* ^2^ tests.

To address the potential confounding effect of differential follow‐up, multivariable linear regression was performed for global satisfaction, KOOS Pain and KOOS QoL, adjusting for graft type, age, sex and follow‐up duration, with RF as the reference category.

Because scar perception may be influenced by patient‐related factors, a multivariable binary logistic regression model was constructed to evaluate whether graft type independently predicted POSAS ≤ 20, adjusting for age and sex.

Odds ratios (ORs) with 95% CIs were reported. Statistical significance was defined as *p* < 0.05. All analyses were performed using SPSS version 27 (IBM Corp.).

The sample size was determined by the primary study from which this cohort was derived. The present analysis of patient‐reported outcomes represents a prospectively planned complementary analysis of the same cohort. Accordingly, the study should be considered exploratory, and results are reported with 95% CIs to allow readers to assess the precision of estimates.

## RESULTS

### Patient characteristics and follow‐up

Of the 90 consecutive patients initially included, one graft failure occurred in the RF group, representing a failure rate of 3.3% (1/30). The affected patient underwent revision surgery before the scheduled outcome assessment and was therefore excluded from the PROM analysis. No graft failures were recorded in the QT or HS groups during the study period. Therefore, 89 patients were available for outcome assessment at a median follow‐up of 15.0 [interquartile range, IQR 13.0–17.0] months, RF 17.0 [IQR 16.0–18.0], QT 14.5 [IQR 14.0–15.2], HS 12.0 [IQR 12.0–15.0] months (*p* < 0.001) (Table 1).

**Table 1:** Baseline demographic and clinical characteristics of the study population.

	Overall (*n* = 89)	RF (*n* = 29)	QT (*n* = 30)	HS (*n* = 30)	*p* value
Age (years)	28.7 ± 8.9	30.9 ± 9.5	27.3 ± 7.8	28.0 ± 9.3	0.226
BMI (kg/m^2^)	24.4 ± 3.3	24.5 ± 3.1	23.8 ± 3.2	24.9 ± 3.7	0.482
Male, *n* (%)	64 (71.9%)	19 (65.5%)	22 (73.3%)	23 (76.7%)	0.621
Right side, *n* (%)	49 (55.1%)	18 (62.1%)	14 (46.7%)	17 (56.7%)	0.482
Meniscal tear, *n* (%)	45 (50.5%)	14 (48.3%)	17 (56.7%)	14 (46.7%)	0.708

Abbreviations: BMI, body mass index; HS, hamstrings autograft; QT, quadriceps tendon autograft; RF, rectus femoris tendon autograft.

### Continuous patient‐reported outcomes

Overall, short‐term patient‐reported outcomes reflected satisfactory postoperative status across the cohort. Mean global satisfaction was 86.5 ± 10.9, KOOS Pain 89.1 ± 13.2, KOOS QoL 71.4 ± 22.2 and EQ‐5D‐5L Index 0.85 ± 0.15.

Global satisfaction did not differ significantly across graft groups (*F* = 2.683, *p* = 0.074). In pairwise comparisons, the RF group showed a mean satisfaction score 6.62 points higher than the HS group (95% CI −0.39 to 13.64, *p* = 0.070).

**Figure 1:**
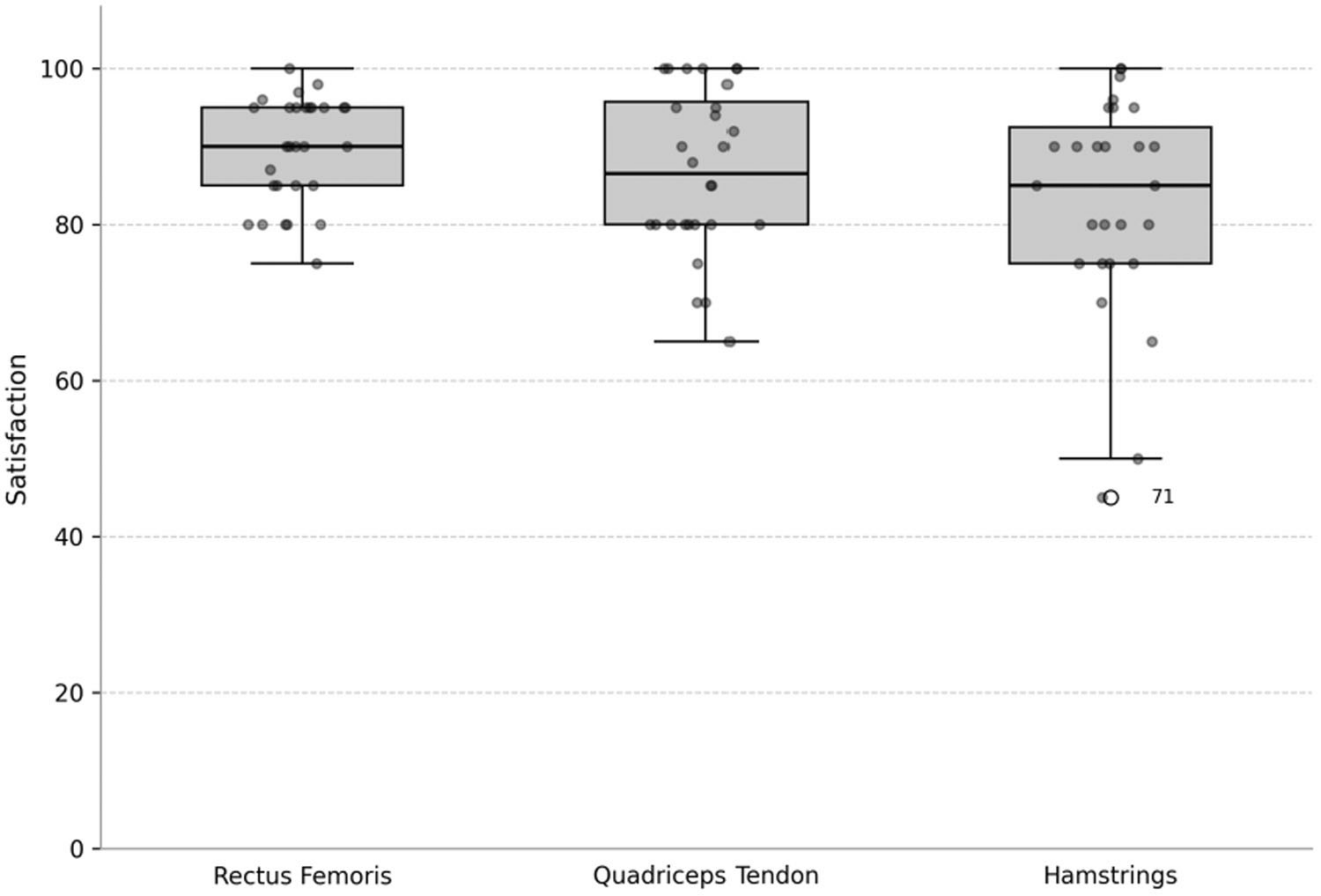
Distribution of global satisfaction at short‐term follow‐up according to graft type.

The distribution of global satisfaction across graft types is illustrated in Figure 1.

**Figure 2:**
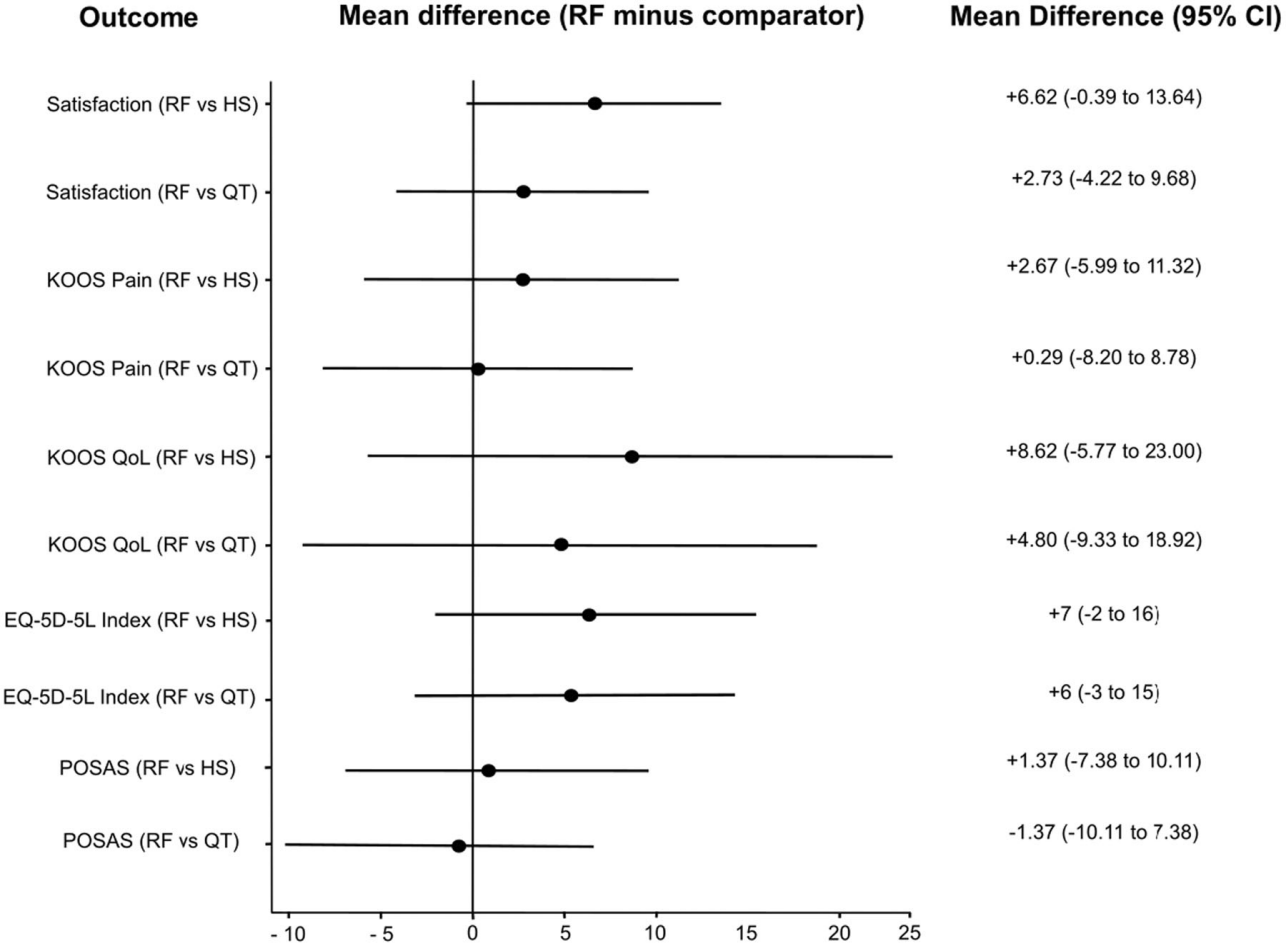
Forest plot comparing rectus femoris (RF) with hamstring tendons (HS) and quadriceps tendon (QT) autografts for satisfaction, KOOS Pain, KOOS Quality of Life (QoL), EuroQol 5‐dimension 5‐level index (EQ‐5D‐5L) and POSAS at short‐term follow‐up. EQ‐5D‐5L Index values were multiplied by 100 for comparability. For POSAS, lower values indicate better scar outcomes; negative differences therefore favour RF. CI, confidence interval; KOOS, Knee injury and Osteoarthritis Outcome Score; POSAS, Patient and Observer Scar Assessment Scale.

No statistically significant differences were observed between graft types in KOOS QoL (*F* = 1.081, *p* = 0.344), KOOS Pain (*F* = 0.335, *p* = 0.716), POSAS (*F* = 0.291, *p* = 0.748) or EQ‐5D‐5L Index (*F* = 0.962, *p* = 0.386). Pairwise mean differences with 95% CIs demonstrating no significant differences between RF and comparator grafts are shown in Figure 2.

In adjusted multivariable analysis accounting for age, sex and follow‐up duration, no statistically significant association between graft type and any primary outcome was observed. For satisfaction: QT *β* = −2.50 (95% CI −8.98 to 3.98, *p* = 0.453), HS *β* = −6.97 (95% CI −14.46 to 0.52, *p* = 0.073). For KOOS Pain: QT *β* = −0.45 (95% CI −8.50 to 7.60, *p* = 0.914), HS *β* = −4.00 (95% CI −13.38 to 5.38, *p* = 0.406). For KOOS QoL: QT *β* = −7.14 (95% CI −21.12 to 6.84, *p* = 0.320), HS *β* = −6.32 (95% CI −22.60 to 9.97, *p* = 0.450).

### High‐threshold outcomes

When applying predefined thresholds, the proportion of patients achieving high satisfaction (≥90) did not differ across graft types (RF: 60.0%, QT: 43.3%, HS: 43.3%; *p* = 0.329). Similarly, KOOS‐Pain ≥90 (RF: 66.7%, QT: 56.7%, HS: 50.0%; *p* = 0.421) and KOOS‐QoL ≥90 (RF: 30.0%, QT: 26.7%, HS: 26.7%; *p* = 0.946) were not significantly different between groups.

### Scar assessment

For scar assessment, the mean POSAS score was 22.3 ± 14.6 in the RF group, 23.7 ± 12.6 in the QT group and 21.0 ± 14.3 in the HS group. The proportion of patients achieving a POSAS outcome ≤20 did not significantly differ in unadjusted analysis (RF: 56.7%, QT: 50.0%, HS: 66.7%; *p* = 0.421). Similarly, after adjustment for age and sex, graft type was not associated with the likelihood of a POSAS score ≤20 (overall model *p* = 0.439). Compared with RF, neither QT (OR 0.84; 95% CI 0.29–2.40) nor HS (OR 1.66; 95% CI 0.56–4.92) showed significant differences. Age (OR 1.05 per year; 95% CI 0.99–1.10) and sex were not independently associated with scar quality within the numbers available.

## DISCUSSION

The principal finding of this study is that no statistically significant differences in short‐term patient‐reported outcomes were observed among patients undergoing ACLR with RF, QT and HS autografts at 1‐year follow‐up. No significant differences were observed in knee‐related QoL, pain perception, global satisfaction or cosmetic scar evaluation at a median follow‐up of 15 months. Overall, these findings do not support a detrimental effect of graft strategy on short‐term postoperative patient‐reported outcomes.

Although graft comparison studies frequently emphasize objective metrics such as graft failure, laxity and strength recovery, these endpoints may not fully capture the subjective postoperative experience at short‐term follow‐up, when patients may be particularly sensitive to residual pain and functional limitation [1, 2, 18, 23, 24, 31]. Patient‐reported outcomes are more commonly assessed at 2 years after ACLR, and comparative data at earlier time points remain relatively limited. Nevertheless, domains such as pain, activities of daily living and health‐related QoL have been shown to reach a functional plateau within the first 12–18 months following ACLR, supporting the clinical relevance of the present follow‐up window, even if longer‐term evaluation of graft performance and return‐to‐sport durability was not possible [25, 27]. The available literature suggests similar early patient‐reported recovery between HS and QT autografts. For instance, Ebert et al. [6] reported similar functional and pain scores at 12 months in a randomized trial comparing HS and QT autografts, and New Zealand registry data likewise demonstrated similar KOOS QoL and pain scores between these graft types at short‐term follow‐up [32]. The present study builds on these findings by demonstrating no statistically significant differences in short‐term pain and knee‐related QoL between HS and QT autografts. Within this exploratory sample, no statistically significant short‐term postoperative disadvantage associated with RF use was detected.

In addition to formal patient‐reported outcomes such as KOOS scores, the absence of statistically significant differences in global satisfaction across groups is noteworthy. Satisfaction reflects the patient's overall appraisal of the surgical result rather than knee symptoms alone, yet it has been reported inconsistently in the ACLR literature. In a systematic review, Kahlenberg et al. [11] found that satisfaction reporting after ACLR was highly heterogeneous and relatively uncommon, with substantial variability in both whether and how satisfaction was measured. As a result, graft‐specific comparisons of satisfaction remain limited. The current findings indicate no statistically significant differences in satisfaction rates across all three graft types within this exploratory sample.

Finally, scar‐related outcomes are rarely reported after ACLR [11]. Among the few comparative studies to specifically examine this domain, Mouarbes et al. [16] evaluated scar cosmesis using the POSAS, a validated tool that assesses both patient‐ and observer‐reported scar characteristics, at 1 year and found no significant difference between QT and HS. The present study is consistent with these prior findings for QT and HS while extending this limited literature by incorporating RF, which likewise did not appear to worsen short‐term patient‐perceived scar outcomes.

Several limitations should be acknowledged. First, per institutional review board requirements, graft allocation was not randomized, and although baseline characteristics did not differ significantly between groups, the possibility of selection bias cannot be fully excluded. The exact distribution of procedures performed by each surgeon across graft groups was not recorded, precluding a formal assessment of surgeon effect on outcomes. Meniscal pathology was recorded as a binary variable only; the type of concomitant meniscal procedure performed was not systematically captured and could not be compared across groups. Furthermore, outcome collection was not blinded to graft type, which may have introduced response bias in patient‐reported measures. Adjusted analysis was restricted to the scar outcome model, which incorporated age and sex as the only available covariates. Other potentially relevant variables were not available for inclusion. Second, and most importantly, the three groups differed not only in graft harvest site but also in surgical construct. The QT group underwent LET through a separate approach using an additional graft, whereas the RF and HS groups achieved anterolateral stabilization percutaneously using a single construct. This distinction means that observed outcomes reflect complete surgical strategies as applied in clinical practice, rather than the isolated effect of graft type alone. Consequently, differences or similarities in patient‐reported outcomes cannot be attributed exclusively to the graft harvest site. While this limits internal validity with respect to graft‐specific conclusions, it enhances the clinical relevance of the comparison, as surgeons selecting among these strategies must consider the full construct, including donor site morbidity, need for additional graft harvest and technical complexity. Third, and critically, preoperative PROMs were not available; therefore, it is not possible to confirm that groups had similar baseline status or to determine whether observed postoperative values reflect similar recovery trajectories. Changes from baseline and achievement of minimal clinically important differences could not be assessed. The present analysis must be interpreted strictly as a cross‐sectional comparison of postoperative patient‐reported status, not as a measure of longitudinal recovery. Fourth, follow‐up was limited to a median of 15 months and differed significantly across graft groups, which may have introduced variability in outcome assessment timing. Long‐term graft performance, structural outcomes and return‐to‐sport durability could not be evaluated. Finally, the sample size was determined by the primary study from which this cohort was derived. No independent power calculation was performed for patient‐reported outcomes, and the study was not adequately powered to detect small between‐group differences, particularly for categorical threshold outcomes. Results should therefore be interpreted as exploratory, with 95% CIs presented throughout to allow assessment of estimate precision.

## CONCLUSION

At 1‐year follow‐up, no statistically significant differences in knee‐related QoL, pain perception, satisfaction or cosmetic scar assessment were observed across the three surgical strategies for ACL reconstruction with concomitant anterolateral procedure. These findings do not provide evidence of a short‐term postoperative disadvantage associated with the RF‐based surgical strategy, though adequately powered studies are needed to draw definitive conclusions.

## AUTHOR CONTRIBUTIONS

**Tomas Pineda**: Formal analysis; writing—original draft. **Nathan H. Varady**: Writing—review and editing. **Grigore Mihai**: Formal analysis; writing—original draft. **Christophe Jacquet**: Supervision; writing—review and editing. **Julien Druel**: Data curation; writing—review and editing. **Matthieu Ollivier**: Supervision; writing—review and editing.

## FUNDING INFORMATION

The authors have no funding to report.

## CONFLICT OF INTEREST STATEMENT

The authors declare no conflicts of interest.

## ETHICS STATEMENT

This study received approval from Aix‐Marseille University, Cse_pads25_041_dgr. Informed consent for research use of the tissues was obtained from all donors or their legal representatives.

## Data Availability

The data sets generated and analysed during the current study are available from the corresponding author upon reasonable request.
